# Synthesis of the Stationary Phase IS-Anionic (Internal Surface-Anionic) for Extraction of Ochratoxin A and B from Samples of Beers

**DOI:** 10.1080/10826070802352827

**Published:** 2008-09-03

**Authors:** M. L. Menezes, E. M. R. S. Simionato, G. Fèlix

**Affiliations:** 1 Universidade Estadual Paulista, Faculdade de Ciências, Departamento de Química, Bauru, São Paulo, Brasil; 2 Universidade do Sagrado Coração – USC, Laboratório de Alimentos, Bauru, São Paulo, Brasil; 3 Laboratoire de Chimie Organique et des Matériaux Moléculaires (CNRS-UMR 6114), Université de la Méditerranée, Marseille cedex, France

**Keywords:** Beer, Internal surface-anionic, Ochratoxin, Stationary phase

## Abstract

A new IS-anionic stationary phase was synthesized to make on-line extraction of the ochratoxin A and B from samples of beer for HPLC. The propyltriethylammonium chloride stationary phase was characterized affecting it’s elementary determination and RI specter, respectively. Evaluation of the IS-anionic column for the extraction and quantification of OTA and OTB in beer has shown that the column is suitable for efficient extraction (recovery >76.5%) and precise analysis. The detection limits for OTA and OTB were 0.03 and 0.07 μgL^−1^, respectively. The range of detector linearity was 0.03 at 20 μgL^−1^.

## Introduction

Ochratoxin A, B, and C are mycotoxins produced by several *Aspergillus* and *Penicilium* species in semitropical and temperate climates.[1] OTA occurs in a variety of food commodities of which cereals and cereal products, fruits, coffee, beer, and wine are the most important sources of intake. OTA is a potent nephrotoxin and hepatotoxin with teratogenic, mutagenic, carcinogenic, and immunosuppressive effects, even at trace levels. In humans, the consumption of OTA contaminated food has been connected to the occurrence of Balkan endemic nephropathy, a disease characterized by severe kidney damage.[2]

Due to its high toxicity and strict regulations on maximum levels of OTA, accurate and sensitive detection is increasingly required, down to the sub-ppb range, in a wide range of different food, feed, and biological matrices.[3,4] Because of the strong native fluorescence activity of OTA, HPLC-FL is now established as the preferred routine analysis technique for OTA, OTB, and OTC and their metabolites, since it offers a better selectivity and sensitivity compared to other detectors.

Usually, solid samples are extracted with water,[5] ethyl acetate,[6] chloroform,[7] methanol-water,[8] acetonitrile-water,[9] and phosphoric acid[6] to enhance solubility and extraction efficiency of OTA.[10] Alternatively, immunoaffinity columns have been applied for cleanup of alimentary extract samples containing mycotoxins. These materials contain immobilized antibodies that exclusively retain OTA, thus producing cleaner extracts with minimum levels of interfering matrix components and excellent signal-to-noise ratios compared to less selective SPE sorbent materials.[1,11] The disadvantage is the high cost, the fact that only some columns exist for some mycotoxins and to the necessity of using a daily pay-column for purification, as the extraction in solid phase SPE.[1] Solfrizzo and collaborators had established a fast method for analysis of OTA in cereals with the use of immunoaffinity columns in the purification of the samples, obtaining lower limits of detection of the method of 1 μg kg^−1^ for 0.21 μg kg^−1^.[12] Chaturvedi and collaborators developed an immunoaffinity column for simultaneous aflatoxin extraction and ochratoxin, aflaochra HPLCTM^TM^ which contains antibodies for aflatoxin and ochratoxin. The mycotoxins are eluted later from the column with methanol and injected into the HPLC.[13] In this work, a method was developed and applied to the extraction of OTA and OTB from samples of beers using a chromatographic column, IS-anionic (internal surface anionic).

## Experimental

### Chemicals and Solvents

Acetonitrile and methanol (HPLC grade) were obtained from Mallinckrodt, (Mallinckrodt of Brazil). The acids, acetic, sulphuric, phosphoric, and potassium dihydrogen phosphate, triethylamine, dimethylformamide, toluene, xylene, and methyl iodide were acquired from Merck (Merck-E, Merck RgaA, Germany). The standards of ochratoxin A and B, human serum albumin, sodium cyanoborohydride, a solution of 25% (ν/ν) glutaraldehyde, silica gel (pore diameter 100Å, particle size 10 μm), and 3-chloropropyltrimetoxisilane, were obtained from Sigma-Aldrich, (Sigma-Aldrich Chemical Company, USA). Pure water was prepared with a Milli-Q system (Millipore, Beldford, MA, USA).

### Preparation of an Ochratoxin A Solution (OTA)

Stock standar solutions were prepared by dissolving known amounts of A ochratoxin in methanol to give solutions containing between 0.015 at 4.0 μg.L^−1^.

All the solutions which contained the mycotoxin were kept frozen at –20°C.

### Preparation of an Ochratoxin B Solution (OTB)

Stock standard solutions were prepared by dissolving known amounts of B ochratoxin in methanol to give solutions containing between 0.015 at 20.00 μg L^−1^.

All the solutions which contained the mycotoxin were kept frozen at –20°C.

### Preparation of the Spiked Beer Sample

Beer was spiked with OTA and OTB by mixing known volumes of the most concentrated solution standard with 30mL of beer, followed by dilution 1:5 with beer:water, thus resulting in beer containing 0.25, 0.50, 1.00, 2.00, and 4.00 μg · L^−1^ and 1.25, 5.00, 10.00, and 20.00 of OTA and OTB, respectively.

### Synthesis of 3-Chloropropyl-Silica Gel

Silica gel, 20 g, (previously activated by heating for 15 hours at 180°C under 1.0 mm Hg pressure), was added to 0.1 mol·L–1 of 3-chloropropyl-trimetoxysilane in a dry 60mL xylene aliquot and the mixture was stirred under reflux for 24 h. After filtration, the product was washed twice with 40mL xylene, followed by acetone. The bonded silica was dried at 120°C under vacuum for 15 h.

### Synthesis of the Stationary Phase, Propyltriethylammonium Chloride

3-Chloropropyl-silica gel 8.0 g, was added to 0.1 mol·L^−1^ of tryethylamine in 50mL of a dimethylformamide aliquot. The mixture was stirred under agitation for 24 hours at room temperature. The same procedure was carried through, keeping the reaction system under reflux for 24 hours at a constant temperature of 60°C. The product was then filtered and was twice washed with 40 mL acetone. The structure of the stationary phase was characterized by IR spectrometry.

### Column Packing

The column was packed by an ascending slurry packing method. The anionic stationary phase was prepared by adding 1.7 g of stationary phase to 60 mL of 0.05 mol·L^−1^ phosphate buffer and sonicated for 5 minutes; it was then quickly transferred to a stainless-steel container used as slurry reservoir, packed into a column (30 mm × 4.6 mm). The packing was performed using a pressure of 300 Bar.

The immobilization of the HAS was performed *in situ* by frontal chromatography in a column packed with the appropriate stationary phase.[14]

### Chromatographic Conditions

#### Instrumentation

The HPLC system consisted of a Varian Model Pro Star Polaris, equipped with two syringe pumps, an electronic control system (MIB), a UV-visible detector, a fluorescence detector set at 325 for excitation and 460 nm for emission, and a data handling station (Workstation-Computer Pentium IV-Dell). On line extraction and separation of proteins and separation of OTA and OTB were carried out by an IS-anionic column (30 mm × 4.6 mm). A manual injector, Rheodyne 7125, (Cotati, CA, the USA), fitted with a 1,000 μL loop was used. The switching-valve (Rheodyne 7000, (Cotati, CA, the USA) was installed to effect the change of flow of the second mobile phase. One used an analytical column Gemini C_18_,5 μm, 110Å, (250 mm × 2.0 mm), installed in series with a Phenomenex column, IS-anionic. It used, as the mobile phase, water (initial mobile phase and 1% HCl: acetonitrile (60:40 ν/ν), with second mobile phase, respectively.

## Results and Discussion

The bonding method used to obtain the stationary phase is very simple. The first step comprised the introduction of the allyl group, followed by the triethylamine group. These were characterized by elementary nitrogen, carbon, and hydrogen analysis, which is shown in Table 1. These values showed that on the surface of the silica gel were present nitrogen, carbon, and hydrogen elements. The 0.35% percentage of nitrogen showed that, on the surface of the silica gel was bonded the triethylamine. The best results were obtained with the stationary phase synthesized at room temperature. From this form, the packing of the chromatographic column with the silica presented a greater carbon percentage. The RI spectrum showed absorption bands at 2,980 and 2,820 cm^−1^ (axial deformation) of the aliphatic C–H and 1,520 and 805 cm–1 (symmetric angular deformation) of the N–H. Two absorption bands at 1,380 and 1,200 cm^−1^ (symmetric deformation and symmetric stretching) were due to the CH_2_ and C–N, respectively. The frequencies of 1,734.540 and 801.349 cm^−1^ were attributed to the quaternary ammonium. These data showed that the propyltriethylammonium chloride group was bonded onto the surface of the silica gel, forming the desired quaternary ammonium stationary phase.

**Table 1:** Values obtained for the elementary analysis of the stationary phases

Stationary phase	Nitrogen (%)	Carbon (%)	Hydrogen (%)
Si0(CH_2_)_3_N^+^(C_2_H_5_)_3_Cl	0.35	11.34	2.43
*Si0(CH_2_)_3_N^+^(C_2_H_5_)_3_Cl^−^	0.30	5.04	1.62

* Synthesis made in the 60°C.

### Evaluation of the Selectivity of the Stationary Phase IS-Anionic

**Figure 1:**
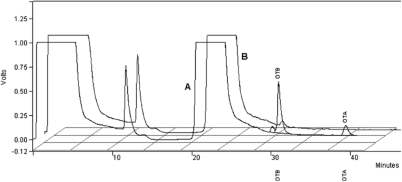
Chromatograms obtained from: (A) after injection of 1,000 μL of 0.8 μg·L^−1^ standard solution of ochratoxins A and B; (B) after injection of 1,000 μL of the beer blank diluted 1:5 (ν/ν). Chromatographic conditions: mobile phase 1-water with the flow-rate 1.0 mL min^−1^ at 10 minutes; after, it was changed to mobile phase 2: 1% aqueous HCl: acetonitrile (60:40 ν/ν); the flow-rate was 0.4 mL min^−1^.

The selectivity of the new stationary phase proposed for OTA and OTB extraction from spiked beer samples was confirmed, comparing the beer chromatogram with the spiked beer chromatogram, as can be seen in Figure 1. In accordance with the chromatogram, we can infer that the developed IS-anionic stationary phase presented selectivity for the studied ochratoxins. The stationary phase, IS-anionic, was installed in series with another chromatographic column, Gemini C_18_ (5 μm particule size, 250 mm × 2.0 mm), with retention times of 31.12 and 40.35 minutes for OTB and OTA, respectively.

The valuation of extraction and separation for OTA and OTB present in spiked beer samples was carried out by direct injection into the IS-anionic column flowing into the C_18_ column. Tables 2 and 3 show the results obtained. The % recoveries were in the range of 78.5 to 91.3 and 76.5 to 93.1; the % CV’s were 5.4 to 11.48 and 4.04 to 10.7 for OTA and OTB, respectively. As can be seen, the IS-anionic column gave good results for extraction of the OTA and OTB present in the spiked beer samples. The extraction of protein using the IS-anionic column carried two reasons: the beer proteins and metabolites not adsorbed by the human serum albumin was immobilized on the external surface of the silica gel and, second, the beer proteins are large molecules that are not able to get into the small silica pores. Various sample solutions were tested for direct injection of beer, but the dilution 1:5 (ν/ν) was better. The newly developed method showed detection limits of 0.03 and 0.07 μgL^−1^ for OTA and OTB, respectively, and good detector linearity was observed within range of 0.03 at 20 μg·L^−1^. The extraction, on-line, of OTA and OTB from spiked beer was shown to be fast and precise, and minimized the time for analysis.

**Table 2:** Recovery (%) and variance coefficient (CV) for extraction of OTA from beer

OTA Spiked level (μg·L^−1^)	OTA Spiked level (1:5) (μg·L^−1^)	OTA detected (μg·L^−1^)	Recovery (%)	Standard deviation	Variance coefficient (%)
		0.033	66.0		
		0.042	84.0		
0.25	0.05	0.039	78.0	9.00	11.48
		0.043	86.0		
			$\bar{X}=78.5$		
		0.087	87.0		
		0.079	79.0		
0.50	0.1	0.076	76.0	4.65	5.75
		0.081	81.0		
			$\bar{X}=80.8$		
		0.197	98.5		
		0.202	101.0		
1.0	0.2	0.173	86.5	9.01	9.78
		0.165	82.5		
			$\bar{X}=92.1$		
		0.327	81.8		
		0.361	90.3		
2.0	0.4	0.365	91.3	4.66	5.36
		0.336	84.0		
			$\bar{X}=86.8$		
		0.672	84.0		
		0.753	94.1		
4.0	0.8	0.714	89.3	6.02	6.59
		0.783	97.9		
			$\bar{X}=91.3$		

**Table 3:** Recovery (%) and variance coefficient (CV) for extraction of OTB from beer

OTB Spiked level (μg·L^−1^)	OTB Spiked level (1:5) (μg·L^−1^)	OTB detected (μg·L^−1^)	Recovery (%)	Standard deviation	Variance coefficient (%)
		0.182	72.8		
		0.192	76.8		
1.25	0.25	0.201	80.4	3.12	4.08
		0.190	76.0		
			$\bar{X}=76.5$		
		0.402	80.4		
		0.482	96.4		
2.50	0.50	0.428	85.6	8.73	10.30
		0.381	76.2		
			$\bar{X}=84.6$		
		0.879	87.9		
		0.881	88.1		
5.0	1.00	0.935	93.5	5.87	6.73
		0.793	79.3		
			$\bar{X}=87.2$		
		1.710	85.5		
		1.576	78.0		
10.0	2.00	1.628	81.4	3.38	4.04
		1.767	88.4		
			$\bar{X}=83.5$		
		4.219	105.5		
		3.591	89.8		
20.0	4.00	3.819	95.5	9.99	10.72
		3.269	81.7		
			$\bar{X}=93.1$		

The newly developed method has been shown to be very simple and precise, when compared with methods for extraction of OTA and OTB mentioned in a review.[15]

## Conclusions

Evaluation of the IS-anionic column for the extraction and quantification of OTA and OTB in beer has shown that the column is suitable for efficient extraction (recovery >76.5%) and precise analysis. On-line extraction is easy because it does not require conventional sample preparation.
